# The impact of COVID-19 on treatment follow-up and medication adherence among patients with epilepsy at a referral hospital in Ethiopia

**DOI:** 10.1371/journal.pone.0299065

**Published:** 2024-02-26

**Authors:** Bethlehem Abera Tekleyohannes, Yared Mamushet Yifru, Beshir Bedru Nasir

**Affiliations:** 1 Department of Pharmacology and Clinical Pharmacy, School of Pharmacy, College of Health Sciences, Addis Ababa University (AAU), Addis Ababa, Ethiopia; 2 Department of Neurology, School of Medicine, College of Health Sciences, AAU, Addis Ababa, Ethiopia; University of the Witwatersrand Johannesburg, SOUTH AFRICA

## Abstract

**Background:**

The world continues to be challenged by the Coronavirus disease 2019 (COVID-19) and preventive measures like maintaining social distancing and lockdowns challenge patients to attend regular follow-ups and get a refill for medication that causes adherence problems. Hence, this study attempts to assess the impact of COVID-19 on treatment follow-up and medication adherence among patients with epilepsy.

**Method:**

A total of 276 patients with epilepsy were enrolled in the study. Data collection was carried out through medical record reviews and patient interviews. Patients who visited Zewditu Memorial Hospital from August to September 2021 and those who had follow-up at least for two years before the outbreak of the pandemic were included. The data was analyzed using SPSS v.24.

**Result:**

About 69.6% of patients were adherent to their treatment and 83.3% of the patients had a seizure-free period of less than 1 year. Ninety (32.6%) of the participants missed their treatment follow-up during the pandemic, mainly due to fear of being infected with COVID-19. Sixty-eight (24.6%) patients have experienced increased seizure episodes during the pandemic as compared to the previous times. Moreover, 56 (20.3%) participants were not taking their antiseizure medications (ASMs) during the pandemic because of the unavailability of medications and they discontinued hospital visits for their medication refills. Among those who missed their treatment follow-up, 20% had seizure-related physical injuries. Only educational level has a significant association with visiting health facilities during the pandemic. Thus, participants who completed college and above (OR = 2.58, 95% CI (1.32–6.38)) were more likely to attend their follow-up during the pandemics as compared to participants who can’t read and write.

**Conclusion:**

The present study revealed that COVID-19 might have impacts on treatment follow-up and medication adherence due to fear of infection, travel restrictions and the indirect impact on the availability and affordability of medications. These might lead to poor treatment outcomes like increased seizure frequency and seizure-related physical injuries.

## Background

Epilepsy is the occurrence of at least two unprovoked (seizures occurring >24 hours apart or one unprovoked seizure with a probability of further seizures risk (at least 60%) occurring over the next 10 years [1]. Individualized pharmacotherapy for patients with epilepsy is highly recommended and treatment regimen selection should also aim to control symptoms and prevent other complications like seizure-related injuries and medication adverse events. Currently, there are several drugs available for the treatment of epilepsy in modern therapy, including newer Antiseizure Medications (ASMs) [2]. However, there are several issues, especially in developing countries, that influence the provision of adequate epilepsy management. The major issues include poor medication adherence, a lack of qualified professionals, the inaccessibility of medications, a lack of knowledge and awareness, a lack of prioritization, and an inadequate health system framework foundation [3].

In both economically developed and developing countries, non-adherence to medication remains a major concern for health care providers and patients because of its negative impact on treatment outcomes. The problem is more significant for patients with chronic diseases that involve complex and long-term treatment regimens [4]. Medication adherence status, especially for chronic diseases like epilepsy, is less than 50% [5]. Non-adherence to ASMs is shown to affect the expected treatment outcomes and it is associated with worsening of disease, death, and increased health care costs [6]. The major reasons for poor adherence to ASMs in Ethiopia include forgetfulness, patients’ feeling as cured, medication unavailability, patients’ beliefs on drug effectiveness, fear of side effects, and switching to traditional medicines [7].

The coronavirus disease 2019 (COVID-19) pandemic was first reported in China in December 2019. Later, on March 11, 2020, the World Health Organization (WHO) pronounced COVID-19 a global pandemic [8] and the Federal Ministry of Health (FMOH) of Ethiopia declared the first case of COVID-19 on March 13, 2020 [9]. The pandemic keeps on having exceptional worldwide effects, forcing an immense strain on the health care system [10]. Moreover, the provision of health services has faced a major obstacle. Thus, preventive measures like maintaining social distancing, self-isolation, avoiding public gatherings and travel restrictions have made it difficult for people to attend regular follow-ups and get refills for their medications, which causes adherence problems [11].

Moreover, coherence in clinical treatment was hindered, which can prompt the progression of the disease and further amplify the treatment difficulties [6]. Similarly, the Ethiopian government took measures such as suspending public gatherings, restricting transportation, closing land borders and declaring a state of emergency [12].

After the COVID-19 outbreak, pharmaceutical companies were busy conducting research and development to control COVID-19. In this regard, low and middle-income countries (LMICs) like Ethiopia might be vulnerable and more affected due to inadequate local pharmaceutical manufacturing capacities to meet their overall drug needs, especially drugs for chronic diseases. With this limited supply and increased demand, the price of medicines for chronic diseases has increased, making them excessively expensive and unaffordable for chronic patients in LMICs who require them [13]. Moreover, adequate treatment outcomes for chronic disease require persistent adherence to medications [14], which is actually related to the accessibility and affordability of medications that can be negatively affected during the outbreak.

Evidence from studies reported that patients experienced a lack of trust in the healthcare system and feared pandemics in a health facility. Thus, patients avoid visiting health facilities for their illness and sending families for medical care, which might increase mortality [15]. Distance from healthcare facilities was shown to decrease utilization of health service delivery during the influenza pandemic. Similarly, the severity of the disease was associated with more health-seeking behavior among individuals with influenza-like illnesses [16].

Specific reasoning for the loss of follow-up (LTFU) and its consequences in the current pandemic at a health care center has not yet been reported in Ethiopia to the best of our knowledge. There is a paucity of data on medication adherence and treatment outcomes for chronic diseases in Ethiopia during the pandemic. Hence, this study attempted to assess treatment follow-up status and adherence to the medications during the pandemic. The findings could be used to uncover practices provided at hospitals, improve services, determine the effect of COVID-19, and serve as a baseline for future studies and a stepping stone for improving adherence in future epidemics.

## Methods

### Study design and period

The study was a hospital-based cross-sectional study among patients with epilepsy. Patients who visited Zewditu Memorial Hospital from August to September 2021 and patients who had follow-up for at least two years before the start of the pandemic were recruited in the study to evaluate medication adherence, follow-up status and treatment outcomes.

### Setting

The study was conducted at Zewditu Memorial Hospital, which is a teaching and general referral hospital affiliated with Tikur Anbessa Specialized Hospital (TASH) of Addis Ababa University. Annually, around 100,000 patients visit the hospital for different health services and the hospital has one hundred fifty-six beds. Services offered at the hospital include cardiac, diabetic, internal pediatrics, medicine, surgery, gynecology and obstetrics, psychiatry, neurology and dermatology.

### Sample size and sampling technique

The sample size was calculated using the single population proportion formula with 50% prevalence and a 95% confidence interval, which gives a sample size of 384. However, the total number of patients with epilepsy who had a follow-up at Zewditu Memorial Hospital was around 720, which is below 10,000. Hence, by using the correction formula and adding a 10% contingency, a total of 276 patients were enrolled. All patients with epilepsy who had a follow-up during the study period and fulfilled the criteria & willing to participate in the study were included. A second visit during the study period (very unlikely) was avoided by asking the participants before the interview.


$$Corrected\;sample\;size=\frac{n\;X\;N}{n+N}$$

N = 720 (total patients with epilepsy at Zewditu Memorial Hospital

n = 384 (possible maximum sample size considering a prevalence of non-adherence of 50%)

Being on ASMs treatment at least two years before COVID-19 and being willing to participate in the study were the inclusion criteria.

### Variables and data collection procedures

The major study variables were medication adherence, treatment outcomes, status of attending treatment follow-up during the pandemic and the presence of seizure-related physical injuries.

The required information about each patient (demographic data and patients’ clinical characteristics, including seizure-related injuries) and status of treatment follow-up during the pandemic and other related data were collected using an interviewer-administered questionnaire.

The questionnaire was first developed in English and then translated into Amharic, then translated back into English by a different person to check its consistency. The questionnaire was pretested on 5% of the sample size before the actual study to check the appropriateness of the data collection tool and assure the completeness of the data. The data used for the pre-test was excluded from the analysis.

Medication adherence was measured using the Morisky Medication Adherence Scale (MMAS-4) Morisky Green Levine test [17] which is available online for free. However, this tool may have low sensitivity and specificity compared to drug levels. A data abstraction form was used to collect pertinent patient information, including the type of seizure and ASMs from medical charts. Treatment outcome was dichotomized based on a previous similar study. Accordingly, patients having a seizure free-period of ≥ 1year were considered as controlled and those having a seizure free-period of <1 year were categorized as uncontrolled seizure [18].

### Data analysis

After cleaning the incomplete the data, the collected data were entered to minimize data entry errors. The data were double entered and checked for discrepancies. Data analysis was carried out using SPSS^®^ Statistics Program Version 23. Descriptive statistics like percentage, frequency, mean and standard deviation (SD) were used to present patients’ characteristics and other related information. The association between the independent variables and follow-up status was checked by binary logistic regression and a *p* value < 0.05 was declared statistically significant.

#### Ethics approval and consent to participate

Ethical approval was obtained from the Ethical Review Board of the School of Pharmacy, College of Health Sciences, Addis Ababa University. A letter of approval was secured from the City Government of Addis Ababa Health Bureau. Before the start of actual data collection, informed written consent was obtained from the study participants and no personal identifier was used. Moreover, the confidentiality of the information obtained from the participants was maintained by keeping the collected data in a secure place.

### Operational definitions

Treatment outcome: means whether a seizure is controlled or uncontrolled.

Controlled seizure: Seizure-free period of ≥ 1year

Uncontrolled seizure: Seizure-free period of <1 year

Unclassified seizure: The diagnosis was documented as epilepsy without a specific seizure type.

Treatment follow-up: patients or caregivers come to the hospital on an appointment date and receive healthcare services like evaluating treatment outcomes and medication refills.

## Results

### Socio-demographic characteristics

Out of the total of 276 study participants enrolled, all were candidates for the analysis. The demographic data revealed that 55.8% of the patients were male. The mean age of the study patients was 22.17 years (SD ± years 15.08). The majority (54.7%) of the study participants have an educational background of primary or secondary school (Table 1).

**Table 1:** Socio-demographic characteristics.

*Variables*		*Number*	*Percentage (%)*
*Sex*	Male	154	55.8%
	Female	122	44.2%
	Total	276	100%
*Age*	Adolescent (<18)	125	45.3%
	Young adult (18–30)	84	30.4%
	Adult (31–60)	63	22.8%
	Elder (>60)	4	1.4%
	Total	276	100%
*Marital status(of those >18 years)*	Single	70	25.4%
	Married	75	27.2%
	Divorced	6	2.2%
	Total	151	54.7%
*Residence*	Rural	23	8.3%
	Urban	253	91.7%
	Total	276	100%
*Educational status*	Unable to read and write	56	20.3%
	Primary school	75	27.2%
	Secondary school	76	27.5%
	University or college	69	25.0%
	Total	276	100%
*Occupation (of those >5years)*	Unemployed	9	3.3%
	Housewife	23	8.3%
	Student	100	36.2%
	Farmer	2	0.7%
	Employed	66	23.9%
	Private	24	8.7%
	Total	224	81.1%

### Clinical characteristics

Out of the 276 participants, only 203 study participants’ charts were reviewed, of which the commonest 134(48.6%) seizure type was Generalized tonic-colonic seizure (GTCS) followed by partial seizure (Table 2). Among the study participants, 186(67.4%) had free medication sources whereas the rest were paying out of pocket.

**Table 2:** Distribution of type of seizure among the study participants.

**Type of seizure**		**Frequency, N (%)**
**Generalized seizure**		*141(69*.*5%)*
	GTCS Atonic seizure Absence seizure	134(66%) 4(1.97%) 3(1.47%)
**Partial seizure**		*35(17*.*2%)*
	Simple partial Complex partial Partial with secondary generalized	27(13.3%) 1(0.4%) 7(3.44%)
**Mixed seizure**		14(6.9%)
Unclassified seizure*		13(6.4%)

*”*Unclassified seizure” was seizure which is diagnosed by the physician as “epilepsy” or “seizure disorder” GTCS-generalized tonic clonic seizure*.

Regarding clinical data, most of the study participants (83.3%) had seizure-free periods of less than a year, and from those not seizure-free, 83% of them had experienced seizure attacks 1–10 times (Table 3).

**Table 3:** Clinical characteristics of the study participants.

Variables		Number	Percent (%)
Seizure since the last visit	No	152	55.1%
	Yes	124	44.9%
	Total	276	100%
Frequency of seizure(since the last visit)	1-5x	104	37.7%
	6-10x	12	4.3%
	>10x	8	2.9%
	Total	124	44.9%
Follow up years	2-5years	186	67.4%
	6-10years	76	27.5%
	>10years	14	5.1%
	Total	276	100%
Seizure free period	<1 year	230	83.3%
	1–5 years	46	16.7%
	Total	276	100%

### The pattern and types of ASMs Use among the study participants

Both monotherapy and combination therapy were used as treatment regimens. Monotherapy 141(69.4%) was the most frequently used treatment regimen and phenytoin (23.1%) was the preferred medication. Dual therapy was also used among 50(24.6%) patients, in which phenytoin plus phenobarbitone combination 17(8.4%) was the most frequently used dual therapy. The rest 7(3.44%) were not taking any medication as it was discontinued and they were just on follow-up (Table 4).

**Table 4:** Type of ASMs prescribed.

Variable		Frequency Percent (%)	
Type AED(s)	PHT	47	23.1%
	PHB	39	19.2%
	VPA	38	18.7%
	CBZ	13	6.4%
	LTG	4	2%
	PHT+PHB	17	8.4%
	VPA+PHB	11	5.4%
	VPA+LTG	5	2.4%
	PHT+CBZ	3	1.5%
	PHT+VPA	3	1.5%
	VPA+CBZ	3	1.5%
	PHB+CBZ	3	1.5%
	PHB+LTG	3	1.5%
	VPA+CLZ	1	0.5%
	PHB+CLZ	1	0.5%
	CBZ+PHB+VPA	1	0.5%
	PHT+PHB+CLZ	1	0.5%
	VPA+LTG+PHB	3	0.5%
	None*	7	3.5%
Total		203	100%

PHB-phenobarbitone VPA-valproic acid CBZ-carbamazepine PHT-phenytoin.

CLZ-clonazepam LTG-lamotrigine.

*those whose medications were tapered down and discontinued, and are just on follow-up.

### Assessment of adherence during COVID-19

Out of the study participants, 90(32.6%) missed their follow-up during the pandemic.

**Fig 1:**
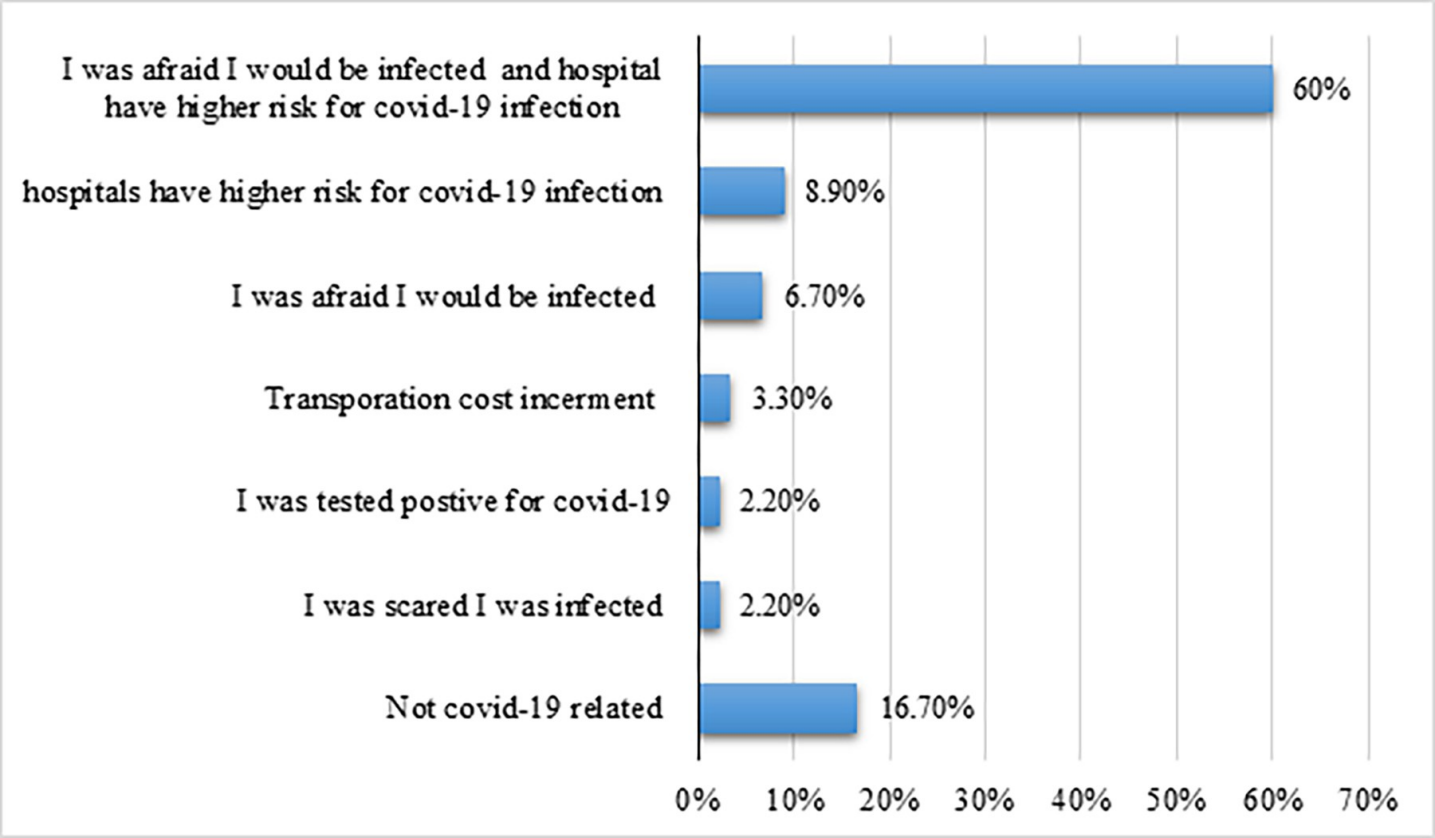
Reason for missing treatment follow-up during the first phase of COVID-19 (April -October 2020).

Among 75(83.3%) of them, the pandemic was the reason for missing their follow-up with different specific reasons (**Fig 1**).

Of the 276 participants, 166(60.1%) agreed that COVID-19 had a negative impact on attending their follow-up, while 97(35.1%) disagreed with the statement and 13(4.7%) were neutral with the statement. Out of those who agreed, 86(51.8%) missed their follow-ups, of which 73(84.8%) were because of the pandemic.

Only education level showed a statistically significant association with follow-up status. Thus, participants with an educational level of college and above (OR = 2.58, 95% CI (1.32–6.38)) were more likely to attend their follow-up during the pandemics as compared to participants who couldn’t write and read (Table 5).

**Table 5:** Factors associated with treatment follow-up during the COVID-19 pandemic.

Variable	Category	Follow-up status		OR (CI 95%)	P-Value
		Yes	No		
Educational status	Unable to read and write	37	19	1.00	
	Primary school	55	20	1.44 (0.84–3.99)	0.082
	Secondary school	63	13	2.42 (0.98–4.22)	0.077
	University or college	59	10	2.58 (1.32–6.38)	**0.038**

### Assessment of treatment outcomes during COVID-19

Out of the 276 participants, only 203 charts were reviewed. The majority 230(83.3%) of the study participants had uncontrolled seizures (seizure-free period less than 1 year); out of these, one-fourth accounts for those who did not attend their treatment follow-up during the pandemic.

Out of 276 participants, 68(24.6%) experienced more seizure episodes and increased seizure frequency during the pandemic compared to previous times. Of those 90 participants who missed their treatment follow-ups, 40 of them had seizure episodes during the time they missed their treatment follow-up. With a seizure frequency of 6(15.0%) had more than ten seizure episodes by the time they missed their appointment, 8(20.0%) had 5–10 seizure episodes, and the rest (65%) had 1–5 episodes.

### Medication adherence during COVID-19

**Fig 2:**
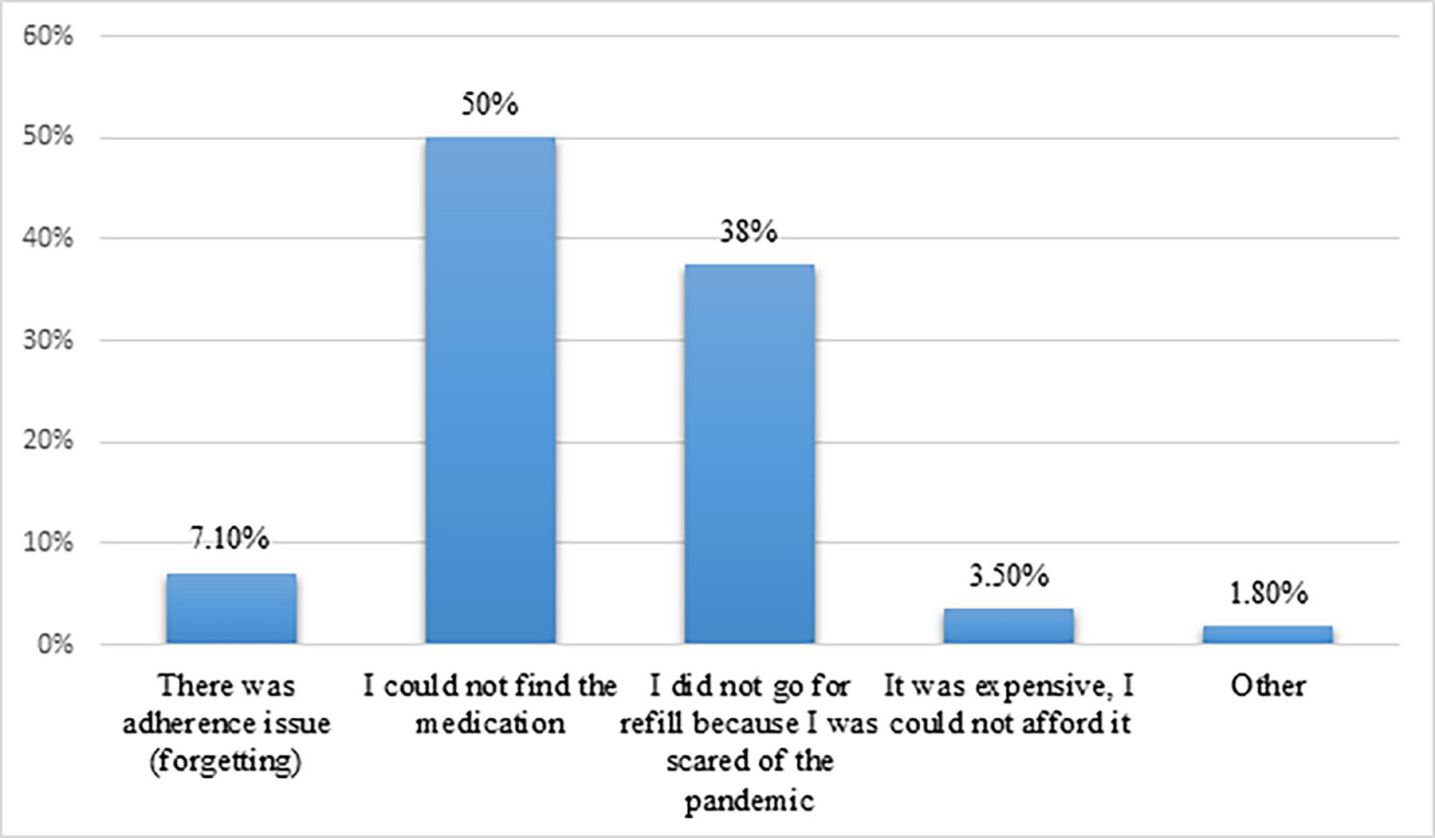
Reasons for non-adherence to medication during the first phase of COVID-19 (April-October 2020).

The MMAS-4 revealed that more than two-thirds 192(69.6%) of the patients were adherent to their medication. The remaining 84(30.4%) were not adherent to their medication. Moreover, out of 276 participants, 56(20.3%) discounted their ASMs during the pandemic because of both the direct and indirect impacts of the pandemic. The major reasons for medication discontinuation were being unable to get the medication and being unable to visit the hospital for refills due to being scared of the pandemic (**Fig 2**).

Of the 276 participants, 214(77.5%) agreed that the COVID-19 pandemic has influenced the availability of their medication and 224(81.1%) agreed that COVID-19 has increased the price of their medications (Table 6).

**Table 6:** The impact of COVID-19 on medication access among the participants.

R. No.	Statements	Agree n(%)	Neutral n(%)	Disagree n(%)
1	COVID-19 influenced availability of my ASMs	214(77.5%)	57(20.7%)	5(1.8%)
2	COVID-19 reduced the affordability /increased the price of my ASMs	224(81.1%)	38(13.8%)	14(5.1%)

#### Prevalence of seizure-related physical injuries

The prevalence of seizure-related physical injuries among study participants who missed their follow-up and had seizure episodes was 8(20%). Of the 20% of seizure-related physical injuries, 1 were dental injuries; 2 were tongue bites; 3 were burns; and 2 were bone fractures.

## Discussion

In this study, 32.6% of the participants missed their treatment follow-up during the pandemic. The finding is in accordance with a pre-post study conducted on changes on clinic visits and admissions at TASH (40.4%) [15] and a similar study conducted on medication adherence among diabetic and hypertensive patients in Addis Ababa, Ethiopia (40%) [19]. But it is lower than the study conducted on the impact of COVID-19 on the care-seeking behavior of patients at TASH (70%) [20]. This discrepancy might be due to a huge gap in the sample size and study participants’ differences, as more than one disease was included in the latter study. However, the numerical finding is highly significant, suggesting that COVID-19 has impaired regular treatment follow-up for chronic disease and imposed a great challenge on optimal treatment outcomes that could have been achieved.

The study revealed that the reasons for missing treatment follow-up were fear of being infected and believing that hospitals have a higher risk for infection (60%) and transport issues (3.03%), which was in concordance with a study conducted at TASH [20].

In this study, sixty-eight (24.6%) of the participants experienced more seizure episodes and increased seizure frequency during the pandemic compared to pre COVID-19, which is in line with a study conducted in Italy (18%) [21]. But it is higher than the finding from a Spanish tertiary hospital (9.8%) [22]. This could be due to differences in patients’ lifestyles when considering the socio-economic status of the countries. Besides, the qualification of expertise and the selection of the most effective ASMs might be the reasons for this discrepancy.

The present study also revealed that 56(20.3%) were not taking their ASMs during the pandemic. This finding was higher compared with a study done in Italy, which was (7%) [22]. This could be due to affordability issues, which were mentioned among the reasons this study was different from other studies. In this study, 80.8% agreed that COVID-19 had an impact on the increased price of their medications and lowered their affordability. This could be a double burden for a developing country like Ethiopia, where adequate health infrastructure and equipment are not met. Moreover, the pandemic-related measures, which include cancellation of flights to certain countries that could affect pharmaceutical importers and a shift of budget to personal protective equipment have exaggerated the price of medications.

The study also revealed that 77.5% agreed that the COVID-19 pandemic has influenced the availability of their medication, which is in accordance with a similar study in which 59% of the participants reported that the local pharmacies could not provide the required medications [20]. As stated above, 80.8% agreed that COVID-19 has increased the price of their medication, which deviates from a study conducted in Addis Ababa, Ethiopia (39%) [19]. This could be due to the fact that most were accessed with community-based insurance coverage before the pandemic, but as availability was compromised during the pandemic, they were forced to pay out of pocket, so the result might have been exaggerated.

The prevalence of seizure-related physical injuries was 20%. Dental injuries, tongue bites, burns, and bone fractures were encountered once. Although the present study lacks detailed data, it is expected that a significant number of patients will die due to severe injuries and injury-related complications in their homes, especially due to car accidents [23].

### Limitations of the study

MMAS-4 is used to assess medication adherence, which may have low sensitivity and specificity.

The seizure type classification was not as per the recent International League against Epilepsy guideline instead an old classification was used because neurologists still use the former classification in clinical practice in the study setting. The number of seizures since the last visit might be ambiguous, as the duration between two visits may not be same for all patients. Moreover, the retrospective nature of the study design might affect the reliability of some data.

## Conclusions

This study revealed that COVID-19 has impacted the treatment follow-up of most of the patients with epilepsy. It also affected patients’ medication adherence due to both the fear of getting infected and the indirect impact it had on the availability and affordability of the medications, which could lead to poor treatment outcomes. Thus, increased seizure frequency and episodes were encountered, as well as transportation problems causing LTFU. Moreover, physical injuries were reported in relation to missed treatment follow-up and increased seizure episodes due to the pandemic. Therefore, a strong patient education program to continue their regular follow-up and availing of the basic medications for epilepsy treatments is recommended for the responsible health facilities.

## Supporting information

S1 File Data collection tool. (DOCX)

## Abbreviations

AAU: Addis Ababa University
ASMs: Antiseizure Medications
CHS: College of health science
FMOH: Federal ministry of health
LTFU: Loss to follow-up
LMICs: Low and middle-income countries
MMAS: 4 Morisky Medication Adherence Scale
SD: Standard deviation
SPSS: Statistical Package for Social Sciences
TASH: Tikur Anbessa specialized hospital
WHO: World Health Organization
