# Supplementary material for: The impact of COVID-19 on treatment follow-up and medication adherence among patients with epilepsy at a referral hospital in Ethiopia
Source: PLoS One. 2024 Feb 26;19(2):e0299065. doi: 10.1371/journal.pone.0299065 (PMC10896525; doi:10.1371/journal.pone.0299065)
Supplement: S1 File — (DOCX) [file pone.0299065.s001.docx]

# Data collection tool (English Version)

**Part I: Socio-demographic Information**

1. **Age**: __________years.
2. **Sex;** Male Female
3. **Marital Status;** Single Married Divorced Widowed
4. **Educational Level:** Unable to read and write Primary school

Secondary school University or college

1. **Place of Residence;** Rural Urban
2. **Occupation;** Unemployed House wife Student Farmer Employed

Other(s) [Specify]:___________________

**Part II Clinical characteristics**

1. When did the first seizure episode occur? ________________

2. When did you start antiepileptic drug(s) treatment? ____________________________

3. When did you encounter the recent seizure episode? _____________________________

4. Was there any seizure since the last visit? Yes or No

5. If yes, how many times? __________________

6. Number of seizure episode in the last one year? ______________

7. Source of medication? Free or Payment

**Part III**

**Assessments of treatment follow up and medication adherence during covid-19 pandemic**

1. Have you been attending your treatment follow ups regularly during the first phase of the pandemic (April – October 2020)? Yes or No
2. If No, what were the reasons?
3. I was afraid that I would be infected
4. Hospitals have higher risk for covid-19 infection
5. I was scared that I was infected
6. I was tested positive for covid-19
7. I could not afford the transportation cost
8. It was not related to the pandemic
9. Others, specify__________
10. Did you have seizure episode at the time you missed your appointment? Yes or No
11. If Yes, how many times?_________
12. If Yes, was there any physical injury related to it?
13. If Yes, state the type of injury
14. Burn
15. Head injury
16. Bone fracture
17. Tongue biting
18. Other, specify______________
19. Did you have more seizure episode after the pandemic compared to the previous times? Yes or No
20. Have you been taking you antiepileptic medication properly during the pandemic? Yes or No
21. If No,

A) There was adherence issue (forgetting)

1. I did not go for refill because I was scared of the pandemic
2. I did not go a pharmacy to buy because I was scared of the pandemic
3. I could not find the medication
4. It was expensive, I could not afford it
5. Other, specify_____________
6. Have you experienced that the COVID-19 has increased seizure frequency or severity? Yes or No

|  | Strongly agree | Agree | Neutral | Disagree | Strongly disagree |
| --- | --- | --- | --- | --- | --- |
| 1. Do you agree that the COVID-19 has negatively affected meeting your follow-up appointments? |  |  |  |  |  |
| 1. Do you agree that the COVID-19 has influenced availability of your chronic care medications? |  |  |  |  |  |
| 1. Do you agree that the COVID-19 has reduced the affordability /increased the price of antiepileptic medications? |  |  |  |  |  |

**Part IV: Assessment of adherence at the current time**

| 1. Morisky Medication Adherence Scale (MMAS-4) | | |
| --- | --- | --- |
| Question | Yes | No |
| - 1. Do you ever forget to take your epilepsy medicine? |  |  |
| 1.2. Do you ever have problems remembering to take your epilepsy medication? |  |  |
| 1.3. When you feel better, do you sometimes stop taking your epilepsy medicine? |  |  |
| 1.4. Sometimes if you feel worse when you take your epilepsy medicine, do you stop taking it? |  |  |

# Appendix III

**Data abstraction format from patient medical records**

9. Type(s) of antiepileptic drug(s) prescribed________________________________________

10. Types of seizure_________________________

# Data collection tool in local language (Amharic Version)

**“የኮቪድ -19 ወረሽኝ ተፅእኖ በመድኃኒት አወሳሰድ እና ክትትል ላይ በዘውዲቱ መታሰቢያ ሆስፒታል የሚጥል በሽታ ሕመምተኞች መካከል”**

**ክፍል 1-ማህበራዊ-ስነ-ሕዝብ መረጃ**

1. ዕድሜ: ______________
2. ፆታ፡ ወንድ/ ሴት
3. የጋብቻ ሁኔታ፡ ያላገባ ያገባ የተፋታች ባል ወይም ሚስት የሞተባት
4. የትምህርት ደረጃ፡ ማንበብ እና መጻፍ የማይችል የመጀመሪያ ደረጃ ትምህርት

ሁለተኛ ደረጃ ትምህርት ዩኒቨርሲቲ ወይም ኮሌጅ

1. የመኖሪያ ቦታ; ገጠር ከተማ
2. የስራ ዘር ፍሙያ; ሥራ አጥ የቤት እመቤት ተማሪ ገበሬ ተቀጣሪ የግል ሌሎች [ይግለጹ]: ___________________

**ክፍል-2 ክሊኒካዊ ባህሪዎች**

1. ለመጀመሪያ ጊዜ ህመሙ የጣሎት/በበሽታው ምክንያት የወደቁት መቼ ነበር? ________________
2. ለህመሙ/ለበሽታው የመድሃኒት ሕክምና መቼ ጀመሩ? ____________________________
3. የቅርብ ጊዜ የመጣል ችግር መቼ አጋጠመው? ____________________________
4. ካለፈው የህክምና ቀጠሮ ጀምሮ የመጣል ችግር ነበር? አዎ ወይም አይ
5. አዎ ከሆነ ፣ ስንት ጊዜ? __________________
6. ባለፈው አንድ ዓመት ውስጥ የመጣል ክስተት ቁጥር ስንት ነበር? ______________
7. የመድኃኒት ምንጭ? ነፃ/ክፍያ

**ክፍል-3 በኮቪድ -19 ወረርሽኝ ወቅት የሕክምና ክትትል እና የመድኃኒት አወሳሰድ ግምገማዎች**

1. ወረርሽኙ መጀመሪያ እንደገባ (መጋቢት - ጥቅምት 2012/2013) የሕክምና ክትትልዎን በመደበኛነት እየተከታተሉ ነበር? አዎ ወይም አይ
2. ካልሆነ (ከአንድ በላይ መምረጥ ይቻላል)

ሀ) በበሽታው እጠቃለሁ ብዬ ፈርቼ ነበር

ለ) ሆስፒታሎች ለኮቪድ -19 ኢንፌክሽን የመጋለጥ እድላቸው ከፍተኛ ነው

ሐ) በበሽታው መያዜን ፈርቼ ነበር

መ) ኮቪድ -19 እንደያዘኝ በምርመራ ተነግሮኝ ነበር

መ) የመጓጓዣ ወጪውን አልቻልኩም

ረ) ከወረርሽኙ ጋር የተገናኘ አልነበረም

ሰ) ሌሎች ፣ ይግለጹ__________

1. ቀጠሮዎን ባመለጦት ጊዜ የመጣል ችግር አጋጥሞዎት ነበር? አዎ ወይም አይ
2. አዎ ከሆነ ፣ ስንት ጊዜ? ________
3. አዎ ከሆነ ፣ ከእሱ ጋር የተያያዘ የአካል ጉዳት ነበር?
4. አዎ ከሆነ የጉዳቱን ዓይነት ይግለጹ(ከአንድ በላይ መምረጥ ይቻላል)

ሀ. ቃጠሎ

ለ. የጭንቅላት ጉዳት

ሐ. የአጥንት ስብራት

መ. ምላስ መንከስ

ሠ. ሌላ ፣ ይግለጹ______________

1. ከበሽታው ወረርሽኝ በኋላ ከቀደሙት ጊዜያት ጋር ሲነፃፀር የበለጠ የመጣል ክስተት አለዎት? አዎ ወይም አይ
2. ወረርሽኙ በተከሰተበት ጊዜ መድሀኒቶን በትክክል ሲወስዱ ነበር? አዎ ወይም አይ
3. ካልሆነ፣(ከአንድ በላይ መምረጥ ይቻላል)

ሀ) በመርሳት ችግር

ለ) ወረርሽኙን ፈርቼ ስለነበር እንደገና ለመቀበል አልሄድኩም

ሐ) ወረርሽኙን ስለፈራሁ ለመግዛት ፋርማሲ አልሄድኩም

መ) መድሃኒቱን ማግኘት አልቻልኩም

ሠ) ውድ ነበር ፣ አቅም አልነበረኝም

ረ) ሌላ ፣ ይግለጹ_____________

1. ኮቪድ-19 የመጣል ድግግሞሽ ወይም ከባድነት እንደጨመረ ያምናሉ? አዎ ወይም አይ

|  | በጣም እስማማለሁ | እስማማለሁ | ገለልተኛ | አልስማማም | በጣም አልስማማም |
| --- | --- | --- | --- | --- | --- |
| 11. ኮቪድ -19 የክትትል ቀጠሮዎችዎን በመከታተል ላይ አሉታዊ ተጽዕኖ አሳድሯል ብለው ይስማማሉ? |  |  |  |  |  |
| 12. ኮቪድ -19 የእርስዎ መድኃኒቶች ማገኝነት ላይ ተጽዕኖ አሳድሮል ብለው ይስማማሉ? |  |  |  |  |  |
| 13. ኮቪድ -19 የመድኃኒቶችን ዋጋ የመጨመር ላይ ተጽዕኖ አሳድሮል ብለው ይስማማሉ? |  |  |  |  |  |

**ክፍል 4 - በአሁኑ ጊዜ መድኃኒትን በታዘዘው መሰረት በአግባቡ ስለመውሰድ ግምገማ**

| 1. ሞሪስኪ” መድኃኒትን በታዘዘው መሰረት በአግባቡ ስለመውሰድ” መለኪያ- 8 | | |
| --- | --- | --- |
| ጥያቄ | አዎ | አይደለም |
| 1.1 የሚጥል በሽታ መድሃኒትዎን መውሰድዎን ይረሳሉ? |  |  |
| 1.2 ሚጥል በሽታ መድሃኒትዎን መውሰድዎን በማስታወስ ላይ ችግሮች አጋጥመውዎት ያውቃሉ? |  |  |
| 1.3 ጥሩ ስሜት ሲሰማዎት አንዳንድ ጊዜ የሚጥል በሽታ መድሃኒትዎን መውሰድ ያቆማሉ? |  |  |
| 1.4 አንዳንድ ጊዜ የሚጥል በሽታ መድሃኒትዎን ሲወስዱ የከፋ ስሜት ከተሰማዎት, መውሰድዎን ያቆማሉ? |  |  |
